# Synergies between RNA degradation and *trans*-translation in *Streptococcus pneumoniae*: cross regulation and co-transcription of RNase R and SmpB

**DOI:** 10.1186/1471-2180-12-268

**Published:** 2012-11-20

**Authors:** Ricardo N Moreira, Susana Domingues, Sandra C Viegas, Mónica Amblar, Cecília M Arraiano

**Affiliations:** 1 Instituto de Tecnologia Química e Biológica, Universidade Nova de Lisboa, Av. da República, Oeiras, 2780-157, Portugal; 2 Unidad de Patología Molecular del Neumococo, Centro Nacional de Microbiología, and CIBER Enfermedades Respiratorias, Instituto de Salud Carlos III. Majadahonda, Madrid, 28220, Spain

**Keywords:** RNA turnover, Post-transcriptional control, Quality control, Transcriptional unit, Non-stop RNA decay

## Abstract

**Background:**

Ribonuclease R (RNase R) is an exoribonuclease that recognizes and degrades a wide range of RNA molecules. It is a stress-induced protein shown to be important for the establishment of virulence in several pathogenic bacteria. RNase R has also been implicated in the *trans-*translation process. Transfer-messenger RNA (tmRNA/SsrA RNA) and SmpB are the main effectors of *trans-*translation, an RNA and protein quality control system that resolves challenges associated with stalled ribosomes on non-stop mRNAs. *Trans*-translation has also been associated with deficiencies in stress-response mechanisms and pathogenicity.

**Results:**

In this work we study the expression of RNase R in the human pathogen *Streptococcus pneumoniae* and analyse the interplay of this enzyme with the main components of the *trans*-translation machinery (SmpB and tmRNA/SsrA). We show that RNase R is induced after a 37°C to 15°C temperature downshift and that its levels are dependent on SmpB. On the other hand, our results revealed a strong accumulation of the *smpB* transcript in the absence of RNase R at 15°C. Transcriptional analysis of the *S. pneumoniae rnr* gene demonstrated that it is co-transcribed with the flanking genes, *secG* and *smpB*. Transcription of these genes is driven from a promoter upstream of *secG* and the transcript is processed to yield mature independent mRNAs. This genetic organization seems to be a common feature of Gram positive bacteria, and the biological significance of this gene cluster is further discussed.

**Conclusions:**

This study unravels an additional contribution of RNase R to the *trans*-translation system by demonstrating that *smpB* is regulated by this exoribonuclease. RNase R in turn, is shown to be under the control of SmpB. These proteins are therefore mutually dependent and cross-regulated. The data presented here shed light on the interactions between RNase R, *trans*-translation and cold-shock response in an important human pathogen.

## Background

The ability of bacteria to sense and adapt to environmental changes is critical to survival. Under stress conditions, prokaryotic cells rapidly adjust their gene expression to deal with a changing environment [1]. RNA molecules provide the dynamic link between DNA-encoded information and protein synthesis. A rapid response to a changing environment involves not only transcriptional but also post-transcriptional regulation [2,3]. mRNA decay is of prime importance for controlling gene expression, and the labile nature of the RNA molecules is critical as it allows a rapid adjustment of proteins levels.

Ribonuclease R (RNase R) is a processive 3’-5’ exoribonuclease that belongs to the RNase II family of enzymes [4-7]. Orthologues have been found in most sequenced genomes [8] and have been implicated in the processing and degradation of different types of RNA, such as tRNA, rRNA, mRNA and the small RNA tmRNA [9-15]. RNase R is the only exoribonuclease able to degrade highly structured RNA molecules and therefore, it is particularly important in the removal of RNA fragments with extensive secondary structures [16]. Cold-shock treatment is a condition which thermodynamically favours the formation of highly structured RNA molecules, and this fact probably leads to the marked increase of RNase R under this stress situation. In fact, *Escherichia coli* RNase R is a general stress-induced protein whose levels are highly upregulated under cold-shock [11,12,17]. Stress resistance and virulence are intimately related since many pathogenic bacteria are challenged with very harsh conditions during the process of infection. Not surprisingly, RNase R has been implicated in the establishment of virulence in a growing number of pathogens. These include *Aeromonas hydrophila*, *Shigella flexneri*, enteroinvasive *E. coli*, and *Helicobacter pylori*[18-21]. This enzyme has also been involved in the quality control of defective tRNA and rRNA molecules [13,22]. Furthermore, *E. coli* RNase R was shown to participate in the maturation of the *transfer*-messenger RNA (tmRNA, also called SsrA) [12], an important small RNA involved in *trans*-translation. In *Pseudomonas syringae* and *Caulobacter crescentus*, degradation of tmRNA was also shown to be dependent on RNase R [23,24]. tmRNA together with SmpB are the main components of the *trans-*translation system, an elegant surveillance pathway that directs deficient proteins and mRNAs for degradation while rescuing stalled ribosomes (for a review see references [25,26]). *Trans*-translation allows bacteria to efficiently respond to a variety of stresses and is required for the viability and for the establishment of virulence in many pathogenic bacteria (reviewed by [25,26]). During *trans*-translation RNase R is the key exoribonuclease involved in the degradation of the faulty mRNAs after the release of the halted ribosomes [2,27]. Moreover, in *E. coli* the stability of RNase R was shown to be regulated by interaction with tmRNA/SmpB, which in turn seems to depend on previous RNase R acetylation [28,29].

In previous studies we have purified and biochemically characterized RNase R from *Streptococcus pneumoniae*[30], an important human pathogen that is one of the leading causes of nosocomial infections. Infections like pneumonia, meningitis or sepsis caused by *S. pneumoniae* place this bacterium among the leading causes of mortality from infectious diseases, affecting especially young children and the elderly. Expression of tmRNA in *S. pneumoniae* have been recently demonstrated [31] and our analysis of the pneumococcal genome revealed that the coding sequence of SmpB is located immediately downstream of the gene encoding RNase R (*rnr*). These observations prompted us to study RNase R expression in this bacterium and to analyse the involvement of this exoribonuclease with the *trans*-translation machinery of *S. pneumoniae*. In this report we show that the pneumococcal *rnr* gene is co-transcribed with the flanking genes *secG* and *smpB* from a promoter upstream of *secG*. This conserved location among Gram-positive bacteria may have a relevant biological meaning. We demonstrate that RNase R expression is induced under cold-stress and that the enzyme levels are modulated by SmpB. Conversely we found that SmpB mRNA and protein levels are under the control of RNase R. This finding uncovers an unsuspected additional connection of RNase R with the *trans-*translation machinery.

## Results

### RNase R levels are regulated by temperature and modulated by SmpB

In a previous work, we have biochemically characterized RNase R, the only hydrolytic exoribonuclease described in *S. pneumoniae*[30], but nothing is known about its expression and regulation. In *E. coli* RNase R was previously described to be modulated in response to different stress situations, namely after cold-shock [11,12,17]. It is also known that RNase R is functionally related with the *trans*-translation system in a wide variety of bacteria [12,23,24,27]. Altogether these observations encouraged us to characterize RNase R expression and study its interplay with the *trans*-translation machinery of *S. pneumoniae*.

**Figure 1:**
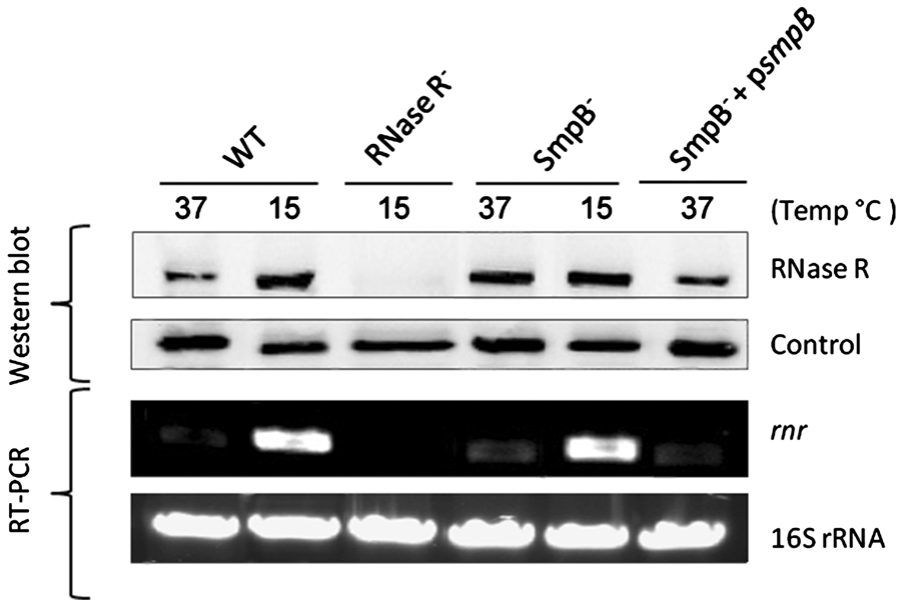
**Pneumococcal RNase R is more abundant under cold-shock and its levels are modulated by SmpB**. Western blot and RT-PCR analysis of protein and RNA samples extracted from wild type and mutant strains as indicated on top of each lane. Details of experimental procedures are described in the ‘Methods’ section. (Upper panel) Analysis of RNase R (~92 kDa) expression by Western blot. RNase R levels were compared in the wild type (WT), the SmpB^-^ mutant and the SmpB^-^ strain expressing SmpB from pLS1GFP at different temperatures (15°C and 37°C). 20 μg of each protein sample were separated in a 7 % tricine-SDS-polyacrylamide gel and blotted to a nitrocellulose membrane. RNase R was detected using specific antibodies. An RNase R^-^ mutant strain was used as a negative control. A non-specific band (Control) detected with the same antibodies was used as loading control. A representative membrane of several independent Western blots is shown. (Lower panel) Analysis of *rnr* mRNA levels by RT-PCR. RT–PCR experiments were carried out with primers specific for *rnr* using 100 ng of total RNA extracted from the wild type (WT) and derivatives at 15°C or 37°C, as indicated on top of the lanes. The RNase R^-^ mutant derivative was used as a negative control. RT-PCR with primers specific for 16S rRNA shows that there were not significant variations in the amount of RNA used in each sample.

To study the expression of RNase R, total protein extracts obtained under physiological temperature and cold-shock were analysed by Western blot using specific polyclonal antibodies that we raised against the purified pneumococcal RNase R. Two hours after a downshift from 37°C to 15°C the protein levels increased around 3-fold (Figure 1). Thus, the expression of the pneumococcal RNase R is modulated by temperature, increasing under cold-shock. In order to determine whether the induction of RNase R could be related with a higher level of the *rnr* transcript under the same conditions, the variation of the *rnr* mRNA levels was evaluated by RT-PCR. A strong increase (~6.5-fold) of the *rnr* transcript was observed under cold-shock (Figure 1). Therefore, the higher levels of RNase R at 15°C are, at least in part, a consequence of the strong increase of the respective mRNA amount.

It has been recently shown that the interaction of SmpB and tmRNA with *E. coli* RNase R destabilizes the ribonuclease [28]. To see if the levels of pneumococcal RNase R were affected by SmpB, comparative Western blot analysis was performed in the presence or absence of SmpB. For this purpose we have constructed an isogenic mutant lacking *smpB* (SmpB^-^) and followed the expression of RNase R at 15°C and 37°C in the wild type, the SmpB^-^ strain, and the SmpB^-^ strain complemented with a plasmid encoding SmpB. As shown in Figure 1, at 15°C the levels of RNase R were roughly the same as in the wild type, but at 37°C there was an increase of the RNase R levels in the SmpB^-^ strain (~2 fold higher than the wild type). The fact that RNase R levels were restored after SmpB expression *in trans*, confirms that SmpB is implicated in the regulation of RNase R. This regulation is probably post-translational, since the *rnr* mRNA levels are roughly the same in the absence of smpB. Interestingly, the effect of SmpB on RNase R is only observed at 37°C. This suggests that the modulation of RNase R by SmpB is probably growth stage-dependent, as it was shown in *E. coli*[29].

Altogether these results indicate that in *S. pneumoniae* SmpB may be one important factor in controlling the levels of RNase R. Nonetheless, the significant increase of the *rnr* mRNA levels under cold-shock may certainly account for the final levels of RNase R in the cell.

### The RNase R transcriptional unit: *rnr* and *smpB* are co-transcribed

**Figure 2:**
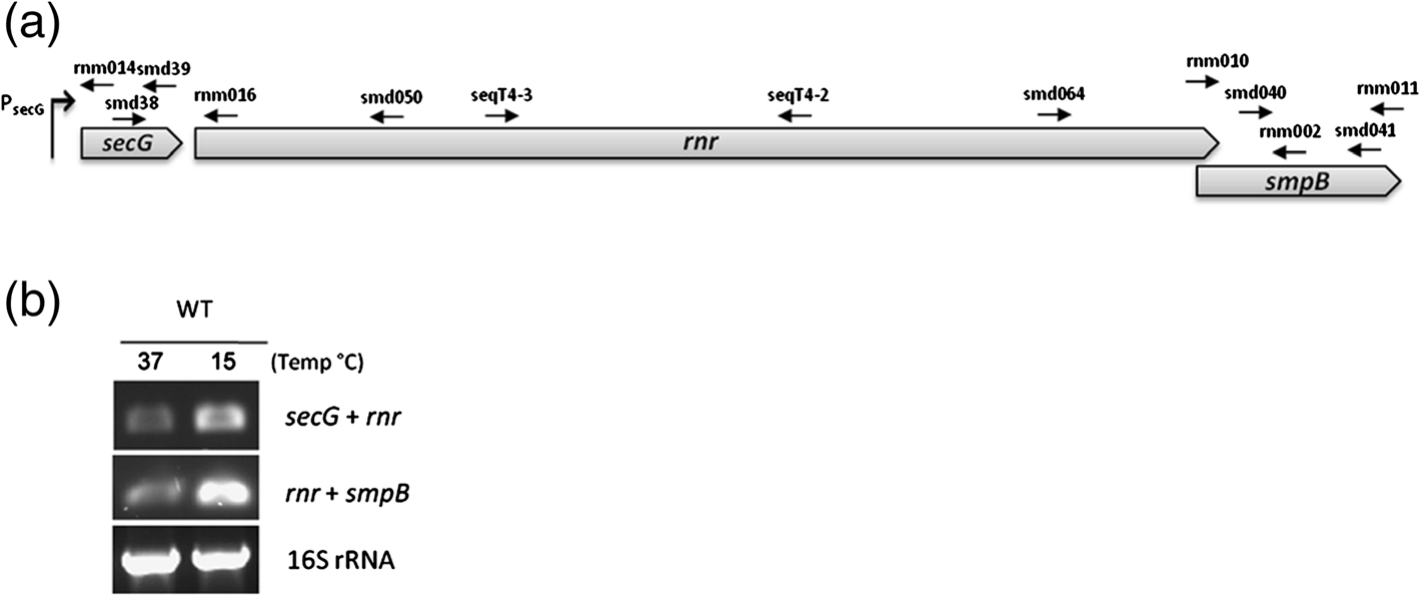
**Analysis of *rnr *transcriptional unit.** (**a**) Schematic representation of the *rnr* transcriptional unit showing the *secG* promoter (P_secG_) identified in this work. The arrows indicate the approximate location and orientation (sense/antisense) of some primers used in RT-PCR, primer extension and RACE experiments. (**b**) *secG, rnr* and *smpB* are co-transcribed. The transcripts were detected by RT-PCR performed with 100 ng of total RNA extracted from the wild type strain at 15°C or 37°C as indicated on top of the lanes. Forward primers annealed to the upstream gene and reverse primers to the downstream gene. Parallel RT-PCR reactions run in the absence of reverse transcriptase yielded no product. RT-PCR with primers specific for 16S rRNA shows that there were not significant variations in the amount of RNA used in each sample.

The cooperation of RNase R and SmpB in important cellular functions, together with the proximal location of their respective coding sequences in the genome of *S. pneumoniae*, led us to further characterize the expression of these two genes. The fact that *rnr* is located upstream and partially overlaps with *smpB* (see Figure 2a) indicates that these genes may be co-transcribed as part of an operon. To check this possibility RT-PCR experiments were carried out using primers smd064 (annealing specifically with *rnr*) and smd041 (annealing specifically with *smpB*) (see localization of primers in Figure 2a). As shown in Figure 2b a fragment that results from the amplification of a transcript containing both *rnr* and *smpB* could be observed, indicating that *smpB* is co-transcribed with *rnr*. A global overview of the *rnr/smpB* genomic region revealed the existence of several ORFs oriented in the same direction (Additional file 1: Figure S1a). The first ORF (a putative metalloprotease represented by “*a”* in Additional file 1: Figure S1a) that is preceded by an ORF oriented in opposite direction is located about 5kb upstream of *rnr*. These ORFs are closely located, some with overlapping regions and, using a specific probe for *smpB* in Northern blot experiments, we detected a high molecular weight transcript (> 8 kb) (Additional file 1: Figure S1b) that could arise from co-transcription of these ORFs. We used the same RT-PCR approach for each pair of consecutive ORFs (using the forward primer to anneal to the upstream ORF and the reverse primer to anneal to the downstream ORF) in order to establish which ORFs were in the same transcriptional unit. A fragment corresponding to the amplification of each ORF pair could be detected (Additional file 1: Figure S1c). The last ORF of this transcriptional unit is most probably *tehB* (“represented by “*k*” in Additional file 1: Figure S1a), since under the same experimental conditions no amplification product containing *tehB* and the downstream gene could be obtained (Additional file 1: Figure S1c, fragment “*k+l*”). In fact, inspection of the sequence revealed a Rho-independent transcription terminator downstream of *tehB.* Interestingly, all the amplification products were detected in a higher amount at 15°C than at 37°C (Additional file 1: Figure S1c) and this is also the case of the high molecular weight transcript detected in the Northern blot experiments (Additional file 1: Figure S1b). These results could be indicative of a cold-shock operon.

**Figure 3:**
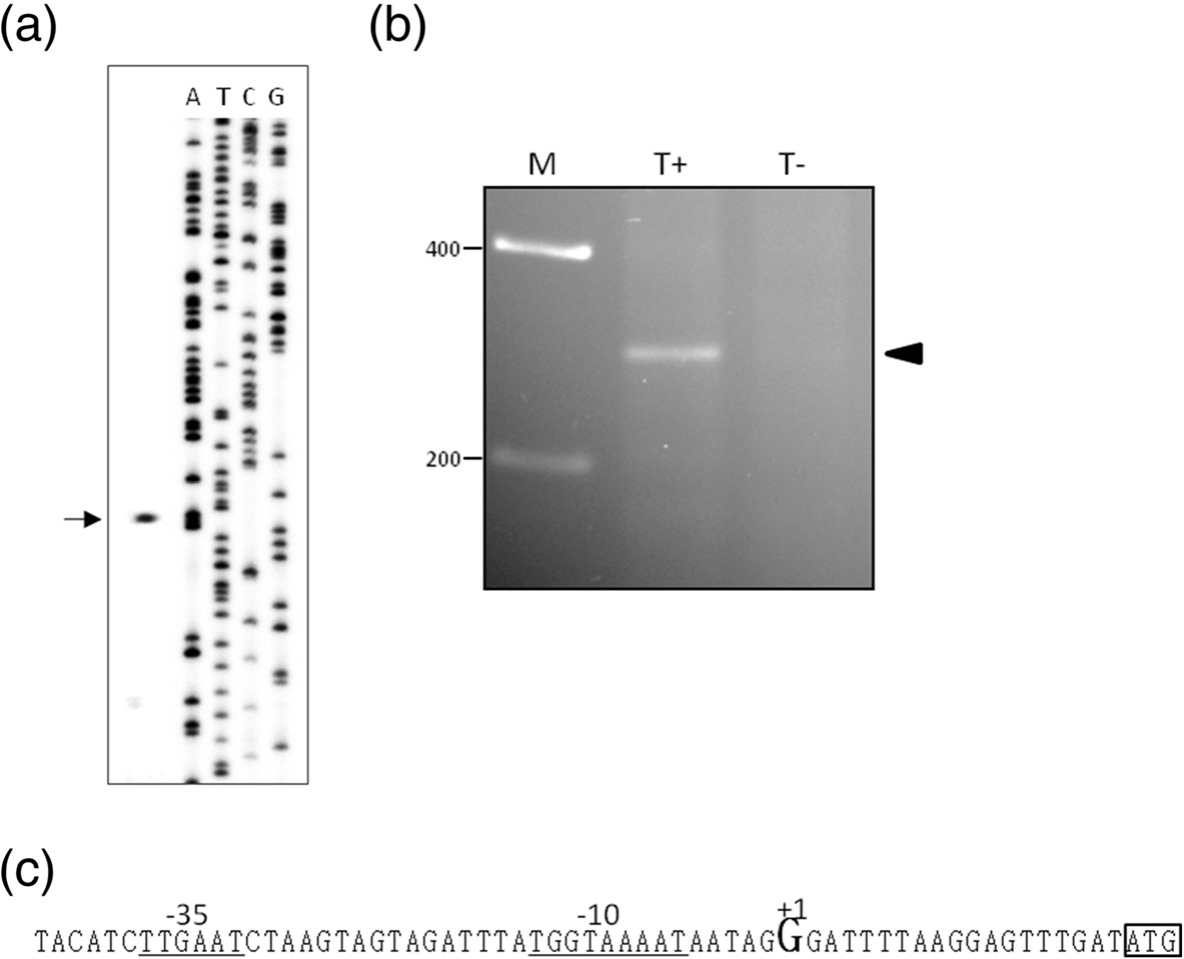
**Mapping of the promoter identified upstream of *secG *(P**_**secG**_**).** (**a**) Primer extension using 5 μg of total RNA extracted from the wild type at 15°C and a 5’-end-labeled primer specific for the 5’region of *secG* (rnm014). The arrow indicates the fragment extended with this primer (128bp). ATCG lanes are sequencing ladders obtained with M13 DNA and a specific radiolabeled primer, which allowed us by size comparison of the unknown product with the ladder to determine the first nucleotide at the 5’-end of *secG* mRNA. (**b**) RACE to map the 5’-end of the *secG* transcript. Reverse transcription was carried out on 6 μg of total RNA extracted from RNase R^-^ strain, using a *secG* specific primer (smd039). PCR signals upon treatment with TAP (lane T+) or without treatment (lane T-) were separated in a 3 % agarose gel. The arrow indicates the signal upon TAP treatment, which corresponds to a primary transcript. Molecular weight marker (Hyperladder I - Bioline) is shown on the left. (**c**) Sequence of the *secG* promoter (P_secG_). The nucleotide corresponding to +1, as determined by primer extension and 5’ RACE, is shown in bold. The −35 and −10 boxes are underlined, and the ATG start codon of *secG* is indicated by a box.

**Figure 4:**
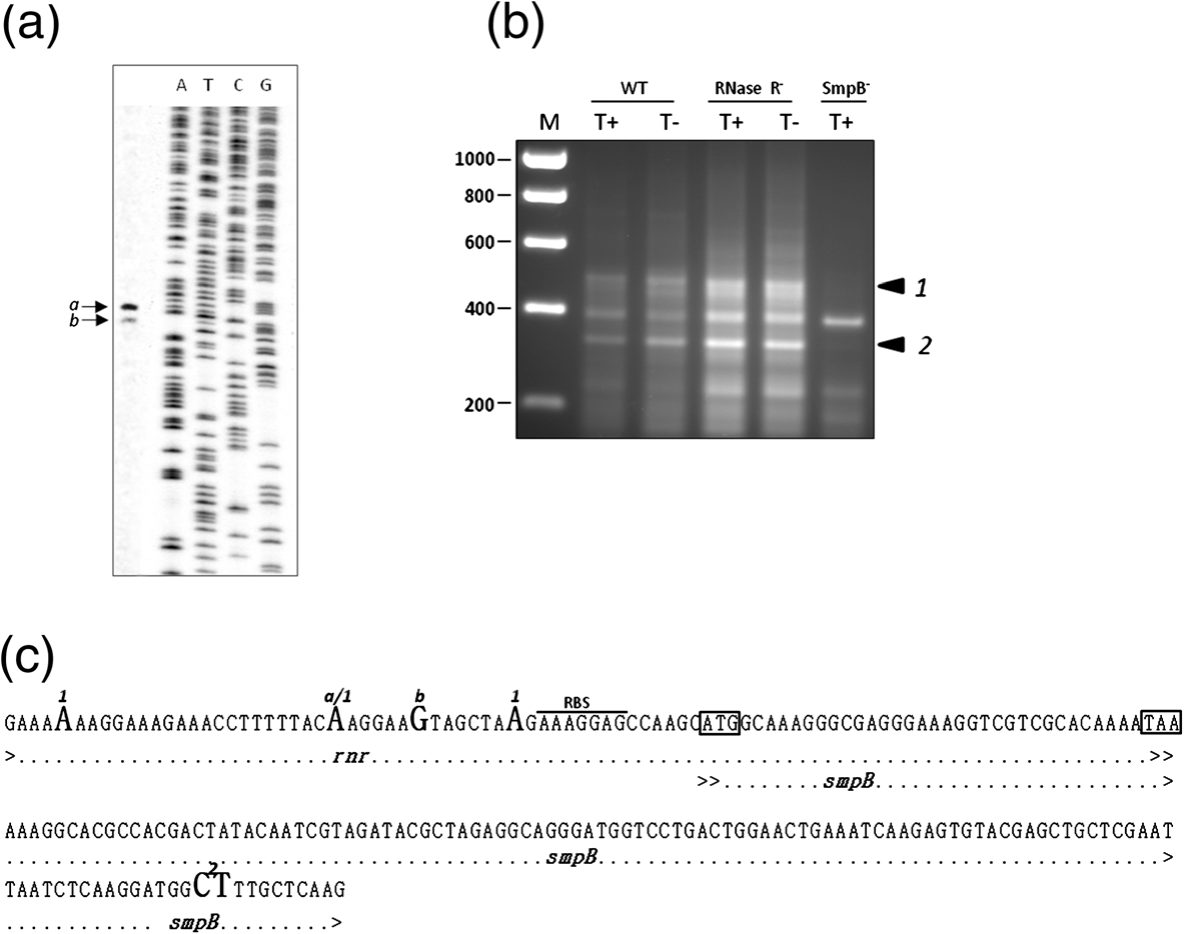
**Primer extension and 5’ RACE analysis of the *rnr *genomic region.** (**a**) Primer extension was carried out with 5 μg of total RNA extracted from the RNase R^-^ strain at 15°C, using a 5’-end-labeled primer specific for the 5’region of *smpB* (rnm002). The arrows indicate the fragments (*a –* 188bp, *b* – 182bp) extended from this primer. The comparison of the fragments sizes with the reading of a generated M13 sequencing reaction provided the determination of the 5’-end of the obtained mRNAs. (**b**) 5’ RACE mapping of the *smpB* transcript. Reverse transcription was carried out on 6 μg of total RNA extracted from wild type and mutant derivatives as indicated on top, using an *smpB* specific primer (rnm011). PCR signals upon treatment with TAP (lane T+) or without treatment (lane T-) were separated in a 3 % agarose gel. As a negative control, the same experiments were performed in the SmpB^-^ strain. The arrows indicate the specific 5’ RACE products (1, 2). Molecular weight marker (Hyperladder - Bioline) is shown on the left. (**c**) Sequence of the region that comprises the 3’end of *rnr* and the 5’end of *smpB*. The nucleotides corresponding to the 5’-end of the extended fragments or to the 5’ RACE products are highlighted in bold. Letters (a, b) or numbers (1, 2) denote primer extension or 5’ RACE results, respectively. The ATG of *smpB* and the stop codon of *rnr* (TAA) are delimited by a dashed box and the putative RBS is indicated.

Taken the above observations a complex regulation of the operon, with multiple promoters and transcripts containing different sets of genes, cannot be ruled out. Since we were particularly interested in *rnr* and *smpB* we have searched for promoters in the vicinity that could regulate the expression of this particular set of genes. Even though bioinformatics analysis indicated a putative promoter immediately upstream of *rnr*, we could not detect any active promoter, either by primer extension analysis or by 5’ RACE mapping (data not shown). Upstream of *rnr* lays a small ORF that encodes a protein with homology to SecG, an auxiliary protein in the Sec-dependent protein export pathway. A transcript containing *secG* and *rnr* was detected and was also mainly expressed under cold shock (Figure 2b). In fact, a putative promoter upstream this ORF was identified *in silico*, which could also drive *rnr* transcription (see Figure 2a). Therefore, primer extension and RACE experiments were conducted to check this possibility. A single fragment was extended from a primer that hybridizes with the 5’-end of the *secG* mRNA (rnm014) as shown in Figure 3a. The size of this fragment, as determined by comparison with the M13 phage sequence, shows that its 5’-end matches the transcription start site (+1) of the *in silico* predicted promoter (see Figure 3c). To confirm this result the 5’-end of the transcript was mapped by 5’ RACE following a protocol that makes use of the tobacco acid pyrophosphatase (TAP) enzyme [32]. This method allows distinguishing between 5’-ends of primary transcripts from those generated by cleavage/processing. A 5’ RACE product that was only obtained from the TAP-treated samples (Figure 3b, lane T+) indicates that it carries a 5’-triphosphate group characteristic of primary transcripts. Cloning and sequencing of this RACE product allowed us to identify the +1 site at the same position as that identified by primer extension. These results clearly show that this promoter is active and drives the expression of *secG*. Considering the lack of a promoter upstream *rnr* and since a transcription terminator could neither be identified in this region, we believe that the *secG* promoter may also contribute to the *rnr* expression. Since our data indicate that *rnr* and *smpB* are co-transcribed, this promoter most likely directs *smpB* transcription as well. Nonetheless, we searched for alternative promoters of *smpB*. We started by analysing the 5’-end of the *smpB* transcript by primer extension using a primer specific for the *smpB* 5’-end region (rnm002 – see Figure 2a). As shown in Figure 4a, two different fragments were extended from this primer (fragment *a* and fragment *b*). Analysis of the sequence revealed that the 5’-ends of both fragments are located right before the overlapping region between *rnr* and *smpB* (Figure 4c). Since by visual inspection we could not identify any putative promoter upstream this region, the extended fragments likely correspond to different processing sites. To clarify the primer extension result and confirm this hypothesis, 5’ RACE experiments were conducted before and after treatment with TAP to discriminate primary transcripts from those originated by processing. The gel in Figure 4b shows several 5’ RACE products that are most probably derived from processed molecules as inferred by the similar intensity of TAP-treated samples. Thereby, under these experimental conditions we did not identify any active promoter upstream *smpB*. This result further corroborates the *rnr* and *smpB* co-transcription hypothesis. The fragments that were not detected in the negative control (Figure 4b, bands 1 and 2) were cloned, and the sequence of several independent clones allowed us to infer the respective 5’-ends. As expected by the smeared-appearance of fragment 1, sequence analysis revealed different transcripts with distinct 5’-ends (Figure 4c). All of these fragments mapped in the 3’-end of *rnr* upstream the overlapping region with *smpB* (Figure 4c), in agreement with the primer extension results. However, only one exactly matched the nucleotide position of one of the extended fragments (Figure 4c, nucleotide signalled “*a/1*”). We do not know the reason for this, but one hypothesis is that these fragments could be the result of trimming by a 5’-3’ exoribonuclease, predicted in this Gram-positive bacterium. Interestingly all the sequences mapped before the putative RBS upstream *smpB* and thus, these processing events may generate a functional independent *smpB* transcript. The sequences of the clones corresponding to the other RACE product (Figure 4b, band 2) mapped inside *smpB* after the overlapping region. While inactivating *smpB* mRNA, this cleavage spares the *rnr* transcript, which may thereby be independently translated.

The fact that the same pattern was obtained from wild type and RNase R^-^ samples (Figure 4b) further confirms that the processing of the *rnr*/*smpB* transcript is not affected in the RNase R^-^ strain.

Taken together these results indicate that the pneumococcal *rnr* transcript is expressed as part of an extensive operon. This large transcript is most probably subject to a complex regulation with several promoters and multiple processing events leading to smaller transcripts. Indeed, a promoter identified upstream *secG* may be responsible for the independent regulation of the downstream genes, *secG*, *rnr* and *smpB*. Processing of the operon to yield mature gene products is likely to occur. Since we could not identify other active promoters upstream *rnr* or *smpB*, we believe that transcription of *rnr* and *smpB* does not occur independently and is most probably driven by the promoter identified upstream of *secG*.

### SmpB mRNA and protein levels are modulated by RNase R

**Figure 5:**
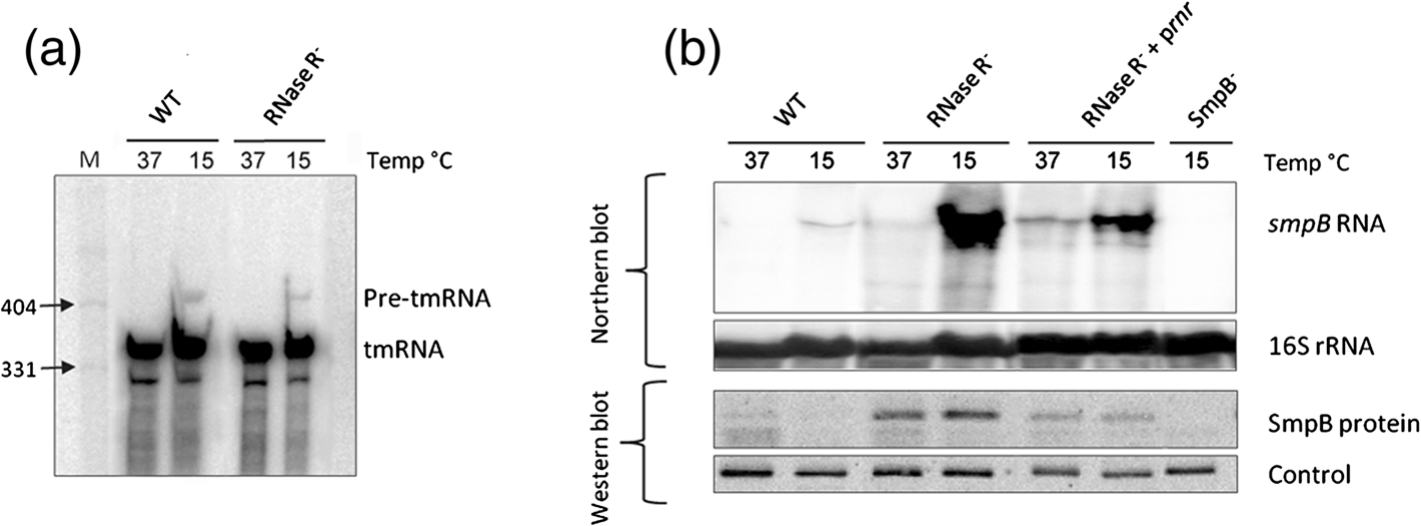
**RNase R regulates SmpB but not tmRNA levels.** Northern blot and Western blot analysis of RNA and protein samples extracted from wild type and mutant strains as indicated on top of each lane. Details of experimental procedures are described in ‘Methods’. (**a**) Analysis of tmRNA by Northern blot. 15 μg of RNA extracted from the wild type (WT) and the RNase R^-^ mutant at 15°C and 37°C were separated on a 6 % polyacrylamide/8.3M urea gel. The gel was then blotted to a Hybond-N^+^ membrane and hybridized with a tmRNA specific riboprobe. (**b**) Analysis of SmpB protein (~18 kDa) and mRNA levels. (Upper panel) 15 μg of total RNA extracted in the same conditions from the wild type, the RNase R^-^ mutant and the RNase R^-^ strain expressing RNase R from pIL253, were separated on a 1.5 % agarose gel, transferred to a Hybond-N^+^ membrane and hybridised with a specific probe for *smpB*. The membrane was stripped and then probed for 16S rRNA as loading control. (Lower panel) SmpB protein levels were analysed by Western blot with SmpB specific antibodies. 20 μg of total protein samples extracted in the same conditions were separated in a 10 % tricine-SDS polyacrylamide gel and blotted to a nitrocellulose membrane. A non-specific band (Control) detected with the same antibodies was used as loading control.

We have just seen that in *S. pneumoniae rnr* is co-transcribed with *smpB*. On the other hand, in *E. coli* SmpB was shown to modulate the stability of RNase R [28]. In *E. coli* processing of tmRNA under cold-shock is dependent on RNase R [12], and this enzyme has also been involved in tmRNA degradation in *C. crescentus* and *P. syringae*[23,24]. Thus, we were interested in clarifying which could be the involvement of RNase R with the main components of the *trans*-translation system in *S. pneumoniae*. For this purpose, we compared both *smpB* and tmRNA expression by Northern blot between the wild type, an isogenic mutant lacking RNase R (RNase R^-^) and the RNase R^–^ strain complemented with a plasmid expressing RNase R constitutively. The results showed that the accumulation of the tmRNA precursor form (pre-tmRNA) at low temperature is similar in the wild-type and the deletion mutant (Figure 5a), and an increase in the tmRNA levels was neither observed in the absence of RNase R. Hence, under our experimental conditions, RNase R from *S. pneumoniae* does not seem to be involved in the tmRNA processing or turnover*.* Nonetheless, analysis of the *smpB* mRNA levels revealed a strong accumulation of the transcript in the absence of RNase R, especially under cold-shock (Figure 5b). Comparison of *smpB* levels between the wild type and the RNase R^-^ strain revealed an increase of about 25-fold at 15°C, while the variation of *smpB* levels at 37°C appeared very low. The lesser accumulation of the *smpB* transcript at 37°C may suggest that in this condition the role of RNase R in the control of this transcript is probably less important. This is in agreement with the low levels of RNase R detected at this temperature (see Figure 1). The involvement of RNase R was further substantiated by complementation of the RNase R^-^ strain with RNase R expressed from pIL253. At 15°C addition of RNase R partially restored the wild type *smpB* levels, leading to a ~17-fold decrease relatively to the RNase R^-^ strain (Figure 5b). Interestingly, in the RNase R complementation strain the variation of *smpB* levels between 15°C and 37°C is lower, suggesting that the temperature-dependent regulation of *smpB* levels is compromised. This is probably due to the fact that RNase R expression from pIL253 is constitutive contrary to the temperature-regulated expression pattern observed in the wild type. Together, these results strongly suggest that RNase R has a role in *smpB* degradation.

To check if the increment observed on the RNA levels would influence the final levels of protein in the cell, we analysed the expression of SmpB under the same conditions. SmpB expression was compared by Western blot in the wild type, the RNase R^-^ mutant derivative and the RNase R^-^ strain complemented with RNase R expressed *in trans*. Analysis of SmpB levels with specific antibodies raised against purified TIGR4 SmpB showed a significant increase in the protein levels in the absence of RNase R (~13-fold at 15°C and ~7-fold at 37ºC) (Figure 5b). This phenotype was partially restored in the strain complemented with RNase R, suggesting that RNase R is determinant for the final levels of SmpB in the cell.

## Discussion

RNase R levels and activity are known to increase in stationary phase and under certain stress situations, namely cold-shock and starvation [11,12,17]. RNase R is the unique exoribonuclease able to degrade RNA molecules with extensive secondary structures, and the increase of RNase R under multiple stress conditions may indicate a general modification of structured RNA in response to environmental changes. In fact this enzyme was shown to be important for growth and viability of several bacteria especially under cold-shock, a condition where RNase R levels are considerably increased [12,18,24,33,34]. Mutants lacking any of the *trans*-translation components (tmRNA and SmpB) also have a variety of stress phenotypes. These range from attenuated antibiotic resistance to problems in adaptation to oxidative stress, cold- and heat-shock [35,36]. In this report we have studied the regulation of the RNase R expression and the interplay of this exoribonuclease with the components of the *trans*-translation system in the human pathogen *S. pneumoniae*.

Our results show that, as occurs in *E. coli,* pneumococcal RNase R is induced after a downshift from 37°C to 15°C. According to our data, both *rnr* mRNA and protein levels are elevated after cold-shock treatment, which could suggest that the higher levels of protein would be directly related with the increased amount of mRNA molecules in the cell. However, the expression of RNase R seems to be also modulated by SmpB. In the absence of this protein the levels of RNase R are similar at 15°C and 37°C and the temperature-dependent regulation observed in the wild type seems to be lost. This result resembles the *E. coli* situation where RNase R was shown to be destabilized by SmpB during exponential phase in a tmRNA-dependent manner [28]. Interestingly, *E. coli* RNase II (a protein from the same family of RNase R) was reported to be destabilized by Gmr, which is encoded by a gene located immediately downstream [37]. Our data suggests that pneumococcal SmpB, which is encoded downstream of *rnr*, may also have a role in the control of RNase R stability. In *E. coli* destabilization of RNase R by SmpB was shown to be dependent on previous acetylation of the enzyme. Acetylation only occurs during exponential growth and was proposed to release the C-terminal lysine-rich region of RNase R [29]. This domain of RNase R is directly bound by SmpB in a tmRNA-dependent manner, and this interaction would ultimately target RNase R for proteolytic degradation [28,29]. We have analysed the pneumococcal RNase R sequence and also identified a lysine-rich C-terminal domain, which could mediate an association between RNase R and SmpB. It seems reasonable to speculate that in *S. pneumoniae*, a similar interaction is taking place.

Interestingly, the lysine-rich domain of RNase R is essential for the enzyme’s recruitment to ribosomes that are stalled and for its activity on the degradation of defective transcripts [38]. A proper engagement of RNase R is dependent on both functional SmpB and tmRNA, and seems to be determinant for the enzyme’s role in *trans-*translation. All these observations point to an interaction between the pneumococcal RNase R and SmpB, which may destabilize the exoribonuclease. However, we believe that the strong increment of the *rnr* mRNA levels detected at 15°C may also account for the final expression levels of RNase R in the cell. A higher amount of mRNA may compensate the low translation levels under cold-shock.

One of the first indications for the involvement of *E. coli* RNase R in the quality control of proteins was its association with a ribonucleoprotein complex involved in ribosome rescue [39]. This exonuclease was subsequently shown to be required for the maturation of *E. coli* tmRNA under cold-shock [12], and for its turnover in *C. crescentus* and *P. syringae*[23,24]. Additional evidences included a direct role in the selective degradation of non-stop mRNAs [2,27] and destabilization of RNase R by SmpB [28]. In this work we strengthen the functional relationship between RNase R and the *trans*-translation machinery by demonstrating that RNase R is also implicated in the modulation of SmpB levels. A marked accumulation of both *smpB* mRNA and SmpB protein was observed in a strain lacking RNase R. The increment in mRNA levels is particularly high at 15°C, the same condition where RNase R expression is higher. This fact suggests that the enzyme is implicated in the control of *smpB* mRNA levels. The higher *smpB* mRNA levels detected at 15°C could also suggest a temperature-dependent regulation of this message. However, the steady state levels of SmpB protein in the RNase R^-^ strain were practically the same under cold-shock or at 37°C. Translational arrest caused by the temperature downshift may be responsible for the difference between the protein and RNA levels. Alternatively, we may speculate that the interaction between RNase R and SmpB could also mediate SmpB destabilization. This hypothesis would imply that RNase R/SmpB protein-protein association would direct both proteins for degradation. Further work is necessary to investigate this attractive possibility.

Analysis of the *S. pneumoniae* RNase R genomic region revealed the presence of several ORFs that may be part of a large transcript shown to be mainly expressed under cold-shock. Some of them are essential for growth, as it is the case of the GTP-binding protein Era and of the Dephospho-CoA kinase. Others are important in the resistance to some drugs or mutagens, as for instance formamidopyrimidine-DNA glycosylase, the multi-drug resistance efflux pump PmrA and the tellurite resistance protein TehB. The first gene of this large operon – YbeY, a putative metalloprotease - appears to be essential for translation under high temperature growth conditions. However, besides RNase R and SmpB none of these genes have known links to cold-stress.

*smpB* is located downstream of *rnr* and we show that both genes are co-transcribed. Although we were not able to identify an active promoter immediately upstream of *rnr* or *smpB* that could drive the transcription of these genes independently, a promoter upstream of *secG* was identified. *secG* is a small ORF located immediately upstream of *rnr* and transcription from its promoter is likely to drive expression of the downstream genes. Indeed, we have demonstrated that this promoter is active and most probably drives the coupled transcription of *secG*, *rnr* and *smpB.*

Identification of processing sites in the overlapping region between *rnr* and *smpB* indicates that this message is processed, yielding either *rnr* or *smpB*. The fact that the coding regions of these genes overlap makes it impossible to have simultaneously both mature mRNAs. Thus, processing of the original transcript always results in disruption of one of the mRNAs. This is in agreement with our results and substantiates the hypothesis of the mutual dependency observed between SmpB and RNase R. In terms of cell physiology it is very interesting to note that when the cell is in need of RNase R and raises its production, the higher amount of enzyme lowers the levels of smpB mRNA. Since SmpB destabilizes RNase R, by lowering the amount of SmpB, the cell guarantees that RNase R will not be degraded. The fact that *smpB* mRNA is disrupted when *rnr* mRNA is matured adds another level of regulation to this complex system. On the other hand when SmpB is required, not only RNase R is destabilized, but its mRNA is also disrupted.

Comparison of the *rnr* genomic region of different Gram-negative and Gram-positive bacteria revealed that this genomic organization (*secG*, *rnr*, *smpB*) seems to be a common feature among Gram-positive bacteria (Table 1). The *rnr* gene is clustered with *secG* and *smpB* in numerous bacteria. Does this close localization have a biological meaning? It is known that bacterial genes involved in the same pathway are frequently co-localized [40]. What could then be the physiological significance of the SecG association with two proteins involved in the *trans*-translation system? SecG is an integral membrane protein that is part of the SecYEG complex involved in the recognition and translocation of appropriate polypeptides through the membrane (see recent reviews [41-43]). Recent data has suggested that *trans*-translation might be linked with other crucial co-translational processes, such as protein folding and secretion [44]. Indeed, problems with folding of nascent polypeptides were recently shown to promote *trans-*translation [45]. This new hypothesis may provide a plausible explanation for the wide array of phenotypes associated with inactivation of tmRNA or SmpB [46]. Most bacterial proteins are secreted through the SecYEG translocator, either during or after translation. When a translocator is blocked in a nascent polypeptide, SecY is degraded, which can be lethal or severely impair cell growth because this protein is required to assemble new translocators [47]. An attractive model for a role of tmRNA in releasing blocked Sec translocators postulates that *trans*-translation activity over a ribosome stalled on a non-stop mRNA during co-translational translocation would allow a tagged protein to be translocated [44]. The subcellular localization of tmRNA and SmpB is also consistent with a link between *tran*s-translation and protein secretion. tmRNA and SmpB are concentrated in a helix-like structure similar to that observed for SecY, SecE, and SecG [48-50]. The close genomic location of *secG*, *smpB* and *rnr* uncovered in this work also points to a functional relationship. This interesting possibility certainly deserves further investigation.

**Table 1:** Organization of the RNase R genomic region in some Gram+ and Gram- bacteria

Gram +	*Streptococcus pneumoniae*	***secG-rnr-smpB***
	*Bacillus subtilis*	***secG****-yvaK-****rnr-smpB****-ssrA*
	*Listeria monocytogenes*	***secG****-LMHCC_0148-****rnr-smpB***
	*Staphylococcus aureus*	***secG****-SAB0735-****rnr-smpB***
	*Clostridium botulinum*	***secG****-****rnr****-surE-****smpB***
	*Lactobacillus acidophilus*	***secG****-****rnr****-****smpB***
	*Enterococcus faecalis*	***secG****-EF2619-EF2618-****rnr****-****smpB***
Gram -	*Escherichia coli*	*nsrR-****rnr****-rlmB-yjfI*^a^
	*Salmonella typhimurium*	*yjeT-purA-yjeB-****rnr****-yjfH-yjfI*
	*Pseudomonas aeruginosa*	***rnr****-PA4936-rpsF*

## Conclusions

In *S. pneumoniae* the RNase R coding region is shown to be part of a large transcript that is mainly expressed under cold-shock. We demonstrate that *rnr* is co-transcribed with the flanking genes- *smpB* (downstream), and *secG* (upstream). A promoter identified upstream of *secG* is likely to control the expression of the downstream genes. Several processing sites in the overlapping region between *rnr* and *smpB* were mapped, indicating that the polycistronic message is processed to yield mature independent mRNAs. The gene cluster “*secG rnr smpB*” appears ubiquitous among Gram-positive bacteria. This finding supports the recently proposed link between *trans*-translation and other crucial co-translational processes, such as protein folding and secretion [44].

This work shows that the expression of the pneumococcal RNase R is modulated by temperature and higher mRNA and protein levels were observed under cold-shock. Additionally it is demonstrated that the *trans*-translation mediator, SmpB, is involved in the regulation of the enzyme expression, leading to increased RNase R levels at 37°C when it is absent. We postulate that in *S. pneumoniae* SmpB may destabilize RNase R at 37°C through a direct protein-protein interaction, as it was shown for *E. coli*[28]. Conversely, a strong accumulation of both *smpB* mRNA and SmpB protein was observed in the absence RNase R. This was mainly observed under cold-shock, the main condition where the RNase R levels are higher. This fact strengthens the role of RNase R in *smpB* degradation at 15°C. The implication of RNase R in the control of SmpB levels reinforces the functional relationship between RNase R and the *trans*-translation machinery, and illustrates the mutual dependency and cross-regulation of these two proteins.

## Methods

### Bacterial growth conditions

*E. coli* was cultivated in Luria-Bertani broth (LB) at 37°C with agitation, unless differently specified. Growth medium was supplemented with 100 μg/ml ampicillin (Amp) when required.

*S. pneumoniae* strains were grown in Todd Hewitt medium, supplemented with 0.5 % yeast extract (THY) at 37°C without shaking, except when differently described. When required growth medium was supplemented with 3 μg/ml chloramphenicol (Cm), 1 or 5 μg/ml Erythromycin (Ery) or 250 μg/ml kanamycin (Km) as specified bellow.

### Oligonucleotides, bacterial strains and plasmids

Unless differently specified all DNA sequencing and oligonucleotide synthesis (Additional file 2: Table S1) were performed by STAB Vida. All PCR reactions to perform the constructions below were carried out with Phusion DNA polymerase (Finnzymes).

*E. coli* strains used in this work are listed in Table 2. All *S. pneumoniae* strains are isogenic derivatives of the JNR7/87 capsulated strain – TIGR4 [51] and are also listed in Table 2.

**Table 2:** List of strains used in this work

**Strain**	**Relevant markers/Genotype**	**Source/Reference**
***E. coli***		
DH5α	F' *fhuA2 Δ(argF-lacZ)U169 phoA glnV44 Φ80 Δ(lacZ)M15 gyrA96 recA1 relA1 endA1 thi-1 hsdR17a*	[52]
CMA601	*E. coli* DH5α carrying pSDA-02	This work
BL21(DE3)	F^–^*omp*T *gal dcm lon hsd*S_B_(r_B_^-^ m_B_^-^) λ(DE3 [*lac*I *lac*UV5-T7 gene 1 *ind*1 *sam*7 *nin*5])	[53]
CMA602	*E. coli* BL21(DE3) overexpressing His-tagged RNase R from *S. pneumoniae* TIGR4	[54]
CMA603	*E. coli* BL21(DE3) carrying pSDA-02	This work
***S. pneumoniae***		
JNR7/87 (TIGR4)		[51]
TIGR4 RNase R^-^	TIGR4 *rnr*^*-*^ (Δ*rnr*-Cm^R^)	C. Arraiano and P. Lopez Labs^a^
CMA604	TIGR4 *rnr*^*-*^ (Δ*rnr*-Cm^R^) carrying pIL253 (Ery^R^) expressing RNase R	This work
CMA605	TIGR4 *smpB*^*-*^ (Δ*smpB*-Kan^R^)	This work
CMA606	TIGR4 *smpB*^*-*^ (Δ*smpB*-Kan^R^) carrying pLS1GFP (Ery^R^) expressing SmpB	This work

^a^ Manuscript in preparation.

The *S. pneumoniae* RNase R^-^ derivative is an in frame deletion of *rnr* that preserves the transcriptional and translational relationships between *smpB* and the upstream ORF. A chloramphenicol-resistance cassette replaces nucleotides +1 to +2288 of the *rnr* gene (Mohedano, Domingues *et al*., manuscript in preparation)

The *S. pneumoniae smpB*^*-*^ deficient mutant was created through allelic replacement mutagenesis [55] using a DNA fragment containing the *smpB* flanking regions, in which *smpB* is replaced by a kanamycin resistance cassette. *km* marker was amplified from pR410 [56] with primers smd019 and smd020. The upstream and downstream *smpB* flanking regions were amplified by PCR using respectively the primer pairs smd053/smd054 and smd055/smd056. Both smd054 and smd055 primers contained 3’ extensions complementary to the 5’- and 3’- ends of the *km* marker, respectively. The combination of these three PCR products was used as template in another PCR reaction performed with primers smd053 and smd056. The resulting PCR product corresponded to a ~3.9 kb fragment containing the *smpB* flanking genes (~1.5 kb each side) and a *km* marker replacing nucleotides +38 to +467 of the *smpB* gene. This fragment was used to transform TIGR4 competent cells of *S. pneumoniae.* Competent cultures of *S. pneumoniae* TIGR4 were prepared in Todd-Hewitt medium (TH) plus 0.5 % glycine and 0.5 % yeast extract by several cycles of dilutions and growing at 37°C up to an OD at 650 nm of 0.3. Competent cells in a concentration 1.5 x 10^7^ CFU/ml were then grown in a casein hydrolase-based medium (AGCH) with 0.2 % sucrose (Suc) and 0.001 % CaCl_2_, and treated with 100 ng/ml of CSP-2 for 14 min at 30°C. Then 590 ng of DNA were added, and the culture was incubated at 30 °C for 40 min. The culture was then transferred to 37°C and incubated for 120 min before plating on media plates (AGCH medium with 1 % agar plus 0.3 % Suc and 0.2 % yeast extract) containing 250 μg/ml Km. Transformants were grown at 37°C in a 5 % CO_2_ atmosphere. A Km^R^ transformant was selected, and the insertion/deletion mutation was confirmed by DNA sequencing at the Genomic Service of Instituto de Salud Carlos III.

In order to express SmpB *in trans*, the TIGR4 SmpB coding sequence was obtained by PCR amplification with primers smd003 and smd004 and was inserted into the unique XbaI site of pLS1GFP [57]). This construction, expressing SmpB from the pneumococcal PM promoter of this plasmid [57], was transformed into the TIGR4 SmpB^-^ strain. Transformants were selected with 1 μg/ml Ery.

The lactococcal plasmid vector pIL253 [58] was used to express TIGR4 RNase R. We have recently shown that this plasmid replicates in *S. pneumoniae* and is suitable for the expression of cloned genes in this bacterium (C. Arraiano, manuscript in preparation). The *rnr* coding sequence was amplified using primers smd093 and smd094 and was inserted into the unique SmaI/PstI sites of pIL253. pIL253 carrying TIGR4 rnr was transformed into *S. pneumoniae* TIGR4 RNase R^-^ and transformants were selected with 5 μg/ml Ery.

*E. coli* SmpB overexpressed in the absence of tmRNA is insoluble [25]. Hence, in order to overexpress and purify pneumococcal SmpB, its coding region was cloned in fusion with pneumococcal *ssrA* (the gene encoding tmRNA) to allow co-expression of both. *smpB* was amplified by PCR with primers rnm010 and rnm011, which contains a 3’ extension complementary to the 5’-end of *ssrA*. *ssrA* was amplified using the primer pair smd057/smd058. The two PCR fragments were then mixed and used as template in a PCR with primers rnm010 and smd058. The resulting PCR product was digested with NdeI and BamHI (Fermentas), and cloned into the pET-15b vector (Novagen) previously cleaved with the same restriction enzymes. This construction, named pSDA-02, was first obtained in *E. coli* DH5α and then transferred to *E. coli* BL21(DE3) to allow the expression of His-SmpB. This construct was confirmed by DNA sequencing.

### Overexpression and purification of proteins

RNase R from *S. pneumoniae* was purified as previously described [30]. For purification of SmpB, BL21(DE3) cells containing pSDA-02 plasmid were grown at 37°C in 250 ml of LB medium supplemented with 100 μg/ml Amp to an OD_600_ of 0.5. Overexpression of SmpB was then induced by addition of 1 mM IPTG; induction proceeded for 3 hours at 37°C. Cells were harvested by centrifugation and stored at −80°C. Purification was performed by histidine affinity chromatography using HisTrap Chelating HP columns (GE Healthcare) and AKTA HPLC system (GE Healthcare) as follows. Frozen cells were thawed and resuspended in lysis buffer (50 mM HEPES pH 7.5, 1 M NH_4_Cl, 5 mM MgCl_2_, 2 mM β-mercaptoethanol, 10 mM imidazole). Cell suspensions were lysed using a French Press at 9000 psi in the presence of 1 mM PMSF. The crude extracts were treated with Benzonase (Sigma) to degrade the nucleic acids and clarified by a 30 min centrifugation at 10000 xg. The clarified extracts were then loaded onto a HisTrap Chelating Sepharose 1 ml column equilibrated with buffer A (20 mM sodium phosphate pH 7.4, 0,5 M NaCl, 20 mM imidazole). Protein elution was achieved by a continuous imidazole gradient (from 20 mM to 500 mM) in buffer A. The fractions containing the purified protein were pooled together and concentrated by centrifugation at 4°C in an Amicon Ultra Centrifugal Filter Device with a molecular mass cutoff of 10 kDa (Millipore). Protein concentration was determined using the Bradford method [59].

SmpB and RNase R purified proteins were loaded in a SDS-PAGE gel and Coomassie blue stained for band excision (data not shown). Bands corresponding to a total of 500 μg of each protein were used to raise antibodies against the respective pneumococcal proteins (Eurogentec).

### RNA extraction and northern blotting

Overnight cultures of *S. pneumoniae* TIGR4 wild type and mutant derivatives were diluted in pre-warmed THY to a final OD_600_ of 0.1, and incubated at 37°C until OD_600_ ~ 0.3. At this point, cultures were split in two aliquots and each aliquot was further incubated at 15°C or 37°C for 2 h. 20 ml culture samples were collected, mixed with 1 volume of stop solution (10 mM Tris pH 7.2, 25 mM NaNO_3_, 5 mM MgCl_2_, 500 μg/ml chloramphenicol) and harvested by centrifugation (10 min, 2800 xg, 4°C). Total RNA was extracted using Trizol reagent (Ambion) essentially as described by the manufacturer, with some modifications. Pneumococcal cells were lysed by incubation in 650 μl lysis buffer (sodium citrate 150 mM, saccharose 25 %, sodium deoxicolate 0.1 %, SDS 0.01 %) for 15 min at 37°C, followed by addition of 0.1 % SDS. After lysis, samples were treated with 10 U Turbo DNase (Ambion) for 1 h at 37°C. After extraction, the RNA integrity was evaluated by gel electrophoresis and its concentration determined using a Nanodrop 1000 machine (Nanodrop Technologies).

For Northern blot analysis, total RNA samples were separated under denaturating conditions either by a 6 % polyacrylamide/urea 8.3 M gel in TBE buffer or by agarose MOPS/formaldehyde gel (1.3 or 1.5 %). For polyacrylamide gels, transfer of RNA onto Hybond-N^+^ membranes (GE Healthcare) was performed by electroblotting (2 hours, 24 V, 4°C) in TAE buffer. For agarose gels RNA was transferred to Hybond-N^+^ membranes by capillarity using 20×SSC as transfer buffer. In both cases, RNA was UV cross-linked to the membrane immediately after transfer. Membranes were then hybridized in PerfectHyb Buffer (Sigma) for 16 h at 68°C for riboprobes and 43°C in the case of oligoprobes. After hybridization, membranes were washed as described [60]. Signals were visualized by PhosphorImaging (Storm Gel and Blot Imaging System, Amersham Bioscience) and analysed using the ImageQuant software (Molecular Dynamics).

### Hybridization probes

Riboprobe synthesis and oligoprobe labelling was performed as previously described [60]. PCR products used as template in the riboprobe synthesis were obtained using the following primer pairs: rnm007/seqT4-3 for *rnr,* T7tmRNA/P2tmRNA for *tmRNA* and smd041T7/smd040 for *smpB*. The DNA probe for 16S rRNA was generated using the primer 16sR labeled at 5’ end with [γ-^32^P]ATP using T4 Polynucleotide kinase (Fermentas).

### Reverse transcription-PCR (RT-PCR)

RT-PCR reactions were carried out using total RNA, with the OneStep RT-PCR kit (Qiagen), according to the supplier’s instructions. The primer pairs seqT4-2/seqT4-3 and rnm010/smd041 were used to analyse *rnr* and *smpB* expression, respectively. Amplification of *secG+rnr* and *rnr+smpB* fragments was performed with the primer pairs smd038/smd050 and smd064/smd041, respectively. The position of these primers in *S. pneumoniae* genome is indicated in Figure 2a. As an independent control, 16S rRNA was amplified with specific primers 16sF/16sR. Prior to RT-PCR, all RNA samples were treated with Turbo DNA free Kit (Ambion). Control experiments, run in the absence of reverse transcriptase, yielded no product.

### Rapid amplification of cDNA ends (RACE) experiments

5’ RACE assays were performed according to Argaman *et al.*[61] with modifications. 5’ triphosphates were converted to monophosphates by treatment of 6 μg of total RNA with 10 units of tobacco acid pyrophosphatase (TAP) (Epicentre Technologies) at 37°C for 30 min in a total reaction volume of 50 μl. The same amount of RNA was used in a parallel reaction where TAP was not added to the sample. To both tubes, 500 pmol of RNA linker and 100 μl of H_2_O were added. Enzyme and buffer were removed by phenol/chloroform/isoamyl alcohol extraction followed by ethanol precipitation. Samples were resuspended in 28 μl of H_2_O and heated-denatured 5 min at 90°C. The adapter was ligated at 4°C for 12h with 40 units of T4 RNA ligase (Fermentas). Enzyme and buffer were removed as described above. Phenol chloroform-extracted, ethanol-precipitated RNA was then reverse-transcribed with gene-specific primers (2 pmol each: smd039 for *secG*; smd050 for *rnr*; rnm011 for *smpB*) using Transcriptor Reverse Transcriptase (Roche) according to the manufacturer’s instructions. Reverse transcription was performed in three subsequent 20 min steps at 55°C, 60°C and 65°C, followed by RNase H treatment. The products of reverse transcription were amplified using 2 μl aliquot of the RT reaction, 25 pmol of each gene specific primer (smd039 for *secG*; smd051 for *rnr*; smd041 for *smpB*) and adapter-specific primer (asp001), 250 μM of each dNTP, 1,25 unit of DreamTaq (Fermentas) and 1x DreamTaq buffer. Cycling conditions were as follows: 95°C/10 min; 35 cycles of 95°C/40 s, 58°C/40 s, 72°C/40 s; 72°C/7 min. Products were separated on 1.5% agarose gels, and bands of interest were excised, gel-eluted (Nucleospin extract: Macherey-Nagel) and cloned into pGEM-T Easy vector (Promega). Bacterial colonies obtained after transformation were screened for the presence of inserts of appropriate size by colony PCR. The plasmids with inserts of interest were purified (ZR plasmid miniprep–classic: Zymo Research) and sequenced.

### Primer extension analysis

Total RNA was extracted as described above. Primers rnm016, rnm014 and rnm002, respectively complementary to the 5’-end of *rnr, secG* and *smpB*, were 5’-end-labeled with [γ-^32^P]ATP using T4 polynucleotide kinase (Fermentas). Unincorporated nucleotides were removed using a MicroSpinTM G-25 Column (GE Healthcare). 2 pmol of the labeled primer were annealed to 5 μg of RNA, and cDNA was synthesized using 10U of Transcriptor Reverse Transcriptase (Roche). In parallel, an M13 sequencing reaction was performed with Sequenase Version 2.0 sequencing kit (USB) using a sequence specific primer, according to the supplier instructions. The primer extension products were run together with the M13 sequencing reaction on a 5 % polyacrylamide / urea 8 M sequencing gel. The gel was exposed, and signals were visualized in a PhosphorImager (Storm Gel and Blot Imaging System, Amersham Bioscience). The size of the extended products was determined by comparison with the M13 generated ladder enabling the 5’-end mapping of the respective transcripts.

### Total protein extraction and western blotting

Cell cultures used to prepare protein extracts were grown in the same conditions as described above for RNA extraction. 20 ml culture samples were collected, mixed with 1 volume of stop solution [10 mM Tris (pH 7.2), 25 mM NaN_3_, 5 mM MgCl_2_, 500 μg/ml chloramphenicol] and harvested by centrifugation (10 min, 2800 xg, 4°C). The cell pellet was resuspended in 100 μl TE buffer supplemented with 1 mM PMSF, 0.15 % sodium deoxicolate and 0.01 % SDS. After 15 min incubation at 37°C, SDS was added to a final concentration of 1 %. Protein concentration was determined using a Nanodrop 1000 machine (NanoDrop Technologies). 20 μg of total protein were separated in a 7 % (for RNase R detection) or 10 % (for SmpB detection) tricine-SDS-PAGE gel, following the modifications described by [62]. After electrophoresis, proteins were transferred to a nitrocellulose membrane (Hybond ECL, GE Healthcare) by electroblotting using the Trans-Blot SD semidry electrophoretic system (Bio-Rad). Membranes were then probed with a 1:1000 or 1:500 dilution of anti-SmpB or anti-RNase R antibodies, respectively. ECL anti-rabbit IgG peroxidase conjugated (Sigma) was used as the secondary antibody in a 1:10000 dilution. Immunodetection was conducted via a chemiluminescence reaction using Western Lightning Plus-ECL Reagents (PerkinElmer).

### Promoter prediction

*In silico* predictions of putative promoters were performed using the BPROM SoftBerry software (http://linux1.softberry.com/berry.phtml?topic=bprom&group=programs&subgroup=gfindb) and Neural Network Promoter Prediction (http://www.fruitfly.org/seq_tools/promoter.html) [63] bioinformatics tools.

## Competing interests

The authors declare that they have not competing interests.

## Authors’ contributions

RNM and SD performed most of the experimental work and drafted the manuscript. SCV did most of the Northern blot analysis and MA made contributions in the construction of mutant strains. CMA supervised the work performed. All authors read and approved the final manuscript.

## Supplementary Material

Additional file 1 **Figure S1** Genomic organization of the *rnr* region in *S. pneumoniae.* Click here for file

Additional file 2 **Table S1** List of oligonucleotides used in this work. Click here for file
