# Supplementary figures and images for: Synergies between RNA degradation and trans-translation in Streptococcus pneumoniae: cross regulation and co-transcription of RNase R and SmpB
Source: BMC Microbiol. 2012 Nov 20;12:268. doi: 10.1186/1471-2180-12-268 (PMC3534368; doi:10.1186/1471-2180-12-268)

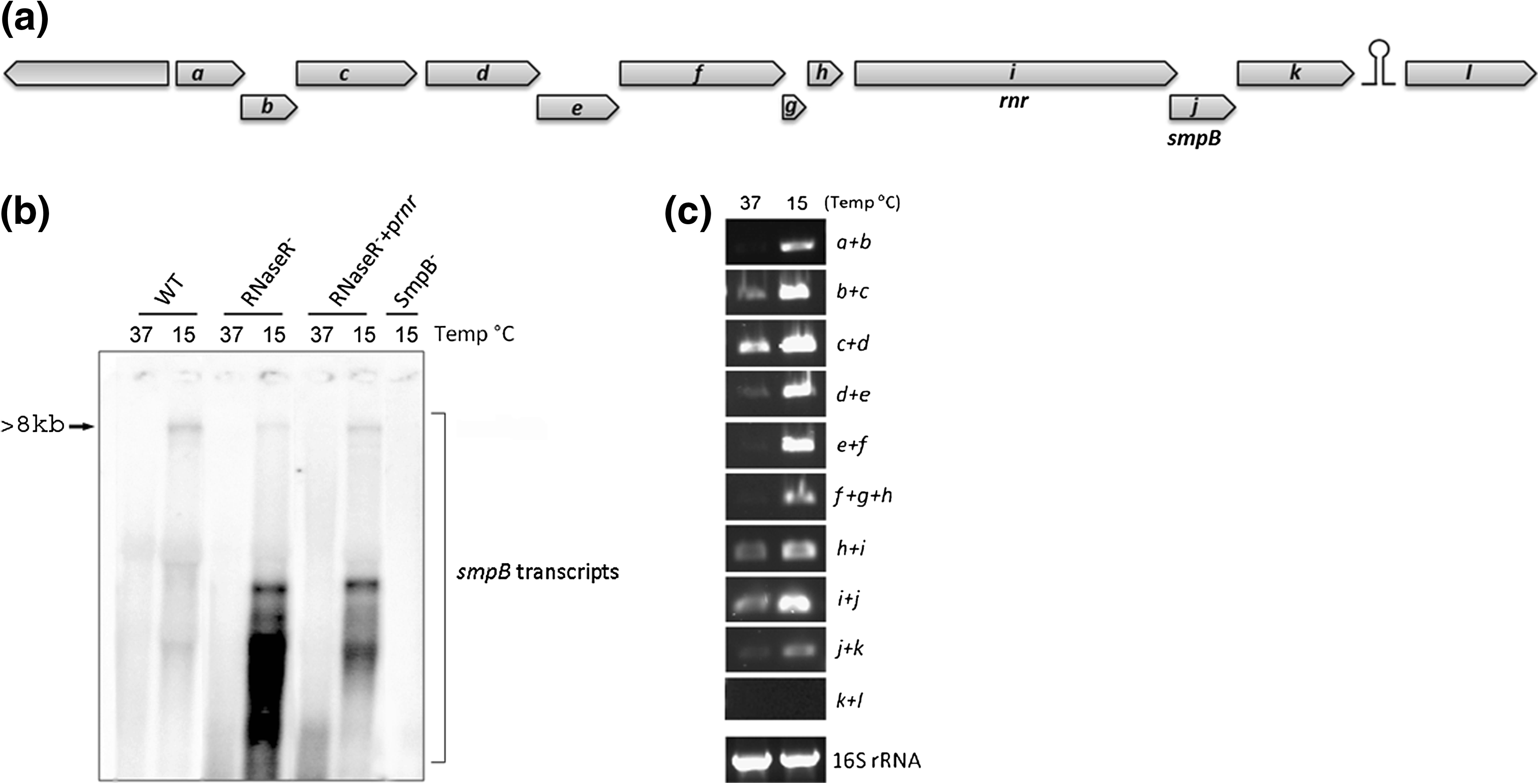

Supplement: Additional file 1 — Figure S1 Genomic organization of the rnr region in S. pneumoniae. [file 1471-2180-12-268-S1.tiff]
