# Supplementary material for: Synergies between RNA degradation and trans-translation in Streptococcus pneumoniae: cross regulation and co-transcription of RNase R and SmpB
Source: BMC Microbiol. 2012 Nov 20;12:268. doi: 10.1186/1471-2180-12-268 (PMC3534368; doi:10.1186/1471-2180-12-268)
Supplement: Additional file 2 — Table S1 List of oligonucleotides used in this work. [file 1471-2180-12-268-S2.docx]

Table S1 – List of oligonucleotides used in this work

| Oligo name | Sequence 5' to 3' |
| --- | --- |
| 16sF | AGAGTTTGATCCTGGCTCAG |
| 16sR | ACGGCTACCTTGTTACGACTT |
| P2tmRNA | GTCGTTACGGATTCGACAG |
| TIGRFw | TCTGGGAGGTGTTGTTGGTC |
| rnm002 | TCACTTGAGCAAAGCCATCC |
| rnm007 | GTTTTTTTTAATACGACTCACTATAGGGACATCGCTATAGGTCATACG |
| rnm010 | GGAATTCCATATGGCAAAGGGCGAGGGAAAGGTC |
| rnm011 | CTAATCTAAAGGCCACTTCCTTATCGCTGATTAACAGCTTTC |
| rnm014 | GAACTGGCATCAAATACATTGCTGGATTGG |
| rnm016 | CCCAAAGCCTGAGCCAAATCATTAACAGTC |
| seqt4-2 | GACATCGCTATAGGTCATACG |
| seqt4-3 | GTTTGACAACAGTTGTCGGG |
| smd003 | GGGGTCTAGAGCTAAGAAAGGAGCCAAGC |
| smd004 | GGGGTCTAGATTTATCGCTGATTAACAGC |
| smd012 | CGCGGATCCAATGAACAGGATTGGTATTG |
| smd019 | GGGCCCGTTTGATTTTTAATG |
| smd020 | GGTACTAAAACAATTCATCC |
| smd033 | TTCTTTCCTTGGGACAATAC |
| smd038 | TTGAACGCAGTAAAGCTCGC |
| smd039 | CTTATCTACTTGATAATACCG |
| smd040 | ATCGTAGATACGCTAGAGGCAGG |
| smd041 | CATCACACGCGCGATATCTC |
| smd041T7 | GTTTTTTTTAATACGACTCACTATAGGCATCACACGCGCGATATCTC |
| smd044 | GAAATGGTAGATGAAACTGG |
| smd050  smd051 | GCTTCTGCTGCTGTTCCCTTATTG  GCGACTTTCTTAATCACTAC |
| smd053 | CGGGGTACCTGGGGTTCACATCGCAGATG |
| smd054 | CATTAAAAATCAAACGGGCCCTTATTTTGTGCGACGACC |
| smd055 | GGATGAATTGTTTTAGTACCAAAGAGGAATTGAAAATGGAAAAATTAG |
| smd056 | CGCGGATCCCCGCGGAGATCCTGGTAAATC |
| smd057 | GGAAGTGGCCTTTAGATTAG |
| smd058 | CGCGGATCCTGGAGCCGGTGGGAGTCGAAC |
| smd064 | CAGTCTAGTCGTAGTGGCAG |
| smd082 | ATGCGCCTGTTAAGGCTGCG |
| Oligo name | **Sequence 5' to 3'** |
| smdo83 | CCATGCTGGCTAAAAATGCC |
| smd084 | GCTACAGAATGCGGAATCTC |
| smd085 | ATCACAGACCATCCAGAACG |
| smd086 | TGTTCGCTTGGTTCAGGACC |
| smd087 | GGTGGCACGCTTGTTTATGAG |
| smd088 | ATAGACCAACCAAGTCTCAG |
| smd089 | GGAGATTATCCGTGAGGAAC |
| smd090 | GCCTTTTCCTTGGCTACTGG |
| smd093 | TTCCCGGGAGAAAGAAAATATGAAAGATAG |
| smd094 | TTTCTGCAGTTTATTTTGTGCGACGACCTTTC |
| T7tmRNA  RNA linker  asp001 | GTTTTTTTTTTAATACGACTCACTATAGGGAGGTGTCTACAACCATAGGTTATG  AUAUGCGCGAAUUCCUGUAGAACGAACACUAGAAGAAA  GCGCGAATTCCTGTAGA |
